# Sarcopenic Obesity Tendency and Nutritional Status Is Related to the Risk of Sarcopenia, Frailty, Depression and Quality of Life in Patients with Dementia

**DOI:** 10.3390/ijerph19052492

**Published:** 2022-02-22

**Authors:** Hsi-Hsien Chou, Te-Jen Lai, Chi-Hua Yen, Po-Sheng Chang, Ji-Cyun Pan, Ping-Ting Lin

**Affiliations:** 1 School of Medicine, Chung Shan Medical University, Taichung 402367, Taiwan; george@csh.org.tw (H.-H.C.); cshy352@csh.org.tw (C.-H.Y.); 2 Department of Neurology, Chung Shan Medical University Hospital, Taichung 402367, Taiwan; 3 Institute of Medicine, Chung Shan Medical University, Taichung 402367, Taiwan; tejenlai@hotmail.com; 4 Department of Psychiatry, Chung Shan Medical University Hospital, Taichung 402367, Taiwan; 5 Department of Family and Community Medicine, Chung Shan Medical University Hospital, Taichung 402367, Taiwan; 6 Department of Nutrition, Chung Shan Medical University, Taichung 402367, Taiwan; s0746002@gm.csmu.edu.tw (P.-S.C.); s0845013@gm.csmu.edu.tw (J.-C.P.); 7 Graduate Program in Nutrition, Department of Nutrition, Chung Shan Medical University, Taichung 402367, Taiwan; 8 Department of Nutrition, Chung Shan Medical University Hospital, Taichung 402367, Taiwan

**Keywords:** nutritional status, sarcopenia, frailty, depression, quality of life, dementia

## Abstract

The purpose of this study was to investigate the nutritional status of dementia patients and examine the correlation with sarcopenia, frailty, depression, and quality of life. We enrolled patients aged 60 years and over with Mini Mental State Examination (MMSE) scores ≤ 26 (Taiwan), and dementia diagnosed by a neurologist or psychiatrist. Nutritional status was assessed with the Mini Nutritional Assessment (MNA). Muscle mass was measured by dual-energy X-ray absorptiometry. Muscle strength and endurance were evaluated by handgrip, leg-back strength, dumbbell curls, sit to stand test, and gait speed. Quality of life, frailty, and depression status were measured by questionnaires. Patients with moderate dementia (MMSE ≤ 20) had a significantly lower MNA score, muscle function, and quality of life than patients with mild dementia (*p* < 0.01). A lower MNA score was significantly associated with the risk of frailty (odds ratio: 4.76, *p* < 0.01), depression (odds ratio: 3.17, *p* = 0.03), and poor quality of life (odds ratio: 2.73, *p* < 0.05), and sarcopenia (odds ratio: 3.97, *p* = 0.03) after adjusting for potential confounders. In conclusion, patients with dementia were at risk of malnutrition, and nutritional status was associated to the risk of sarcopenia, frailty, depression, and quality of life.

## 1. Introduction

The number of deaths from dementia has increased globally. The latest report of the World Health Organization (WHO) indicates that dementia was ranked as the 7th leading cause of death in 2019 [1]. Aging is one of the risk factors for dementia [2]. Throughout Asia, societies have aged, including in Taiwan. A report from Lin and Huang [3] indicated that the aging rate in Taiwan is more than twice that in European countries and the United States, and the number of people with dementia is increasing in Taiwan. According to the report from Taiwan Alzheimer Disease Association indicated that the prevalence of dementia in Taiwanese who aged ≥65 years is 7.71%, which means about 1 in 12 people over the age of 65 have dementia, and about 1 in 5 people who aged ≥80 [4].

Dementia is a term used for a group of symptoms with deterioration in cognition, memory, thinking, emotion, and the ability to perform daily activities [5]. Dementia may lead patients to forget to eat, lose their appetite, or have difficulty preparing meals, which then affects their nutritional status, increasing the incidences of institutionalization and hospitalization, at cost to society [6,7].

Sarcopenia and frailty are also aging-related conditions [8,9]. Individuals with sarcopenia or frailty frequently show a decline in physical function, associated with lower skeletal muscle mass, low muscle strength and endurance, balance dysfunction, and gait variability, as well as falls and fractures [10,11]. A prospective study on community-dwelling elderly individuals in Japan found that sarcopenia-related factors other than aging were cognitive impairment in both men and women and depressed mood in women [12]. Sarcopenia and frailty may also be related to increased depression and psychologically affect their quality of life [13,14]. Most of the previous literature has revealed that sarcopenia is associated with cognitive impairment [15,16,17]. Recently, the new concept of sarcopenic obesity (a combination of sarcopenia and obesity) has emerged and is considered a public health risk in older adults [18,19]. However, few studies have discussed sarcopenic obesity in the dementia population. Additionally, nutritional status is one of the modifiable factors that are involved in the progression of dementia, it is worth assessing nutritional status in patients with dementia. Thus, the purpose of this study was to investigate nutritional status and examine the correlation between nutritional status and sarcopenia, frailty, depression, and quality of life in dementia.

## 2. Materials and Methods

### 2.1. Study Design and Subjects

This study was a cross-sectional study. The subjects were recruited in outpatient from Chung Shan Medical University Hospital between August 2019 and July 2021. Eligible subjects were ≥60 years old with a Mini Mental State Examination (MMSE) score ≤26 and dementia diagnosed by a neurologist or psychiatrist. The cutoff point for MMSE score set at 26 was according to the definition of the Dementia Diagnostic Manual of Taiwan Ministry of Health and Welfare [20]. The exclusion criteria were as follows: (1) patients who were diagnosed with cancer, severe heart, lung, liver, and kidney disease; and (2) severe disability or aphasia. Patients were stratified by severity of dementia, with mild dementia indicated by an MMSE score >20 and moderate dementia indicated by an MMSE score ≤20 [21,22]. This study was approved by the Institutional Review Board of Chung Shan Medical University Hospital, Taiwan (CSMUH No: CS2-18147). Each subject provided informed consent before participating in the study. A total of eighty dementia patients were recruited and completed the analysis for the study.

### 2.2. Demographic Data and Anthropometric Assessments

A questionnaire was used to collect the demographic data of the subjects, including age, gender, education, lifestyle habits, and family history of dementia, which were performed by the research assistant from asked the subjects or their caregiver. Blood pressure was measured by a digital electronic sphygmomanometer (Hartmann Tensoval^®^ duo control, Heidenheim, Germany). Body mass index (BMI) was calculated by height and weight. Waist circumference and calf circumference (CC) were measured by a tape. We used a dual-energy X-ray absorptiometry machine (Hologic, ASY-05119, Marlborough, MA, USA) to measure whole body lean soft tissue, appendicular skeletal muscle mass index (ASMI), and body fat percentage. The definition of obesity was based on the value of body fat percentage ≥25% for males or ≥30% for females [23].

### 2.3. Blood Sample Collection and Hematological Measurements

Vacutainers with sodium fluoride and without anticoagulant (Hebei Xinle Sci&Tech Co., Ltd., Xinle, China) were used to collect fasting venous blood specimens. Serum samples were prepared after centrifugation at 4 °C and 3000 rpm for 15 min. Hematological data, such as albumin, creatinine, glutamic pyruvic transaminase, glucose, and total cholesterol, were measured by an automated chemistry analyzer (Roche, Cobas c501, Rotkreuz, Switzerland).

### 2.4. Nutritional Status, Frailty, Depression and Quality of Life Measurements

The Mini Nutritional Assessment (MNA) was used to estimate the nutritional status of subjects. The MNA questionnaire consisted of 18 questions, including appetite, weight loss, mobility, physical and mental status, and dietary intake. The maximum total score of the screening tool is 30, and a score lower than 24 indicates malnutrition or risk of malnutrition [24]. Frailty was evaluated by the Study of Osteoporotic Fractures (SOF) questionnaire [25]. The SOF questionnaire consisted of 3 domains, including frailty, falls, and depression assessment (6 questions). A higher score of the SOF indicated the poor frailty status. The Geriatric Depression Scale [26] was used to assess the depression level. Higher score of geriatric depression indicate that subjects suffer from depression. The Quality of Life in Alzheimer’s Disease scale was used to assess subjects’ quality of life [27]. A higher quality of life score on the Alzheimer’s Disease scale represents a better quality of life. All questionnaires were performed by the research assistant from asked the subjects or their caregiver.

### 2.5. Muscle Strength and Endurance Assessments

Tests of handgrip and leg-back strength were used to evaluate the upper and lower limb muscle strength, respectively. Handgrip strength was assessed by a handgrip dynamometer (TAKEI, TKK-5401, Niigata, Japan). Subjects were instructed to squeeze the device with the dominant hand as hard as they could. Leg-back strength was assessed by a back dynamometer (TAKEI, TKK-5402, Niigata, Japan). For the test, subjects stood on the base of the back dynamometer; knees and hips flexed slightly while the lower back was maintained upright, and grasped a bar attached to a chain and dynamometer. Then, subjects were asked to lift up the bar with maximal effort. Two measurements were taken with handgrip and leg-back strength tests, and the maximum measurement value was recorded for the analysis.

The dumbbell curls, sit to stand test, and gait speed were used to assess the upper and lower limb muscle endurance according to the Senior Fitness Test Manual [28]. Subjects were asked to flex and extend the elbow with their dominant hand holding an 8 lb dumbbell (for male patients) or a 5 lb dumbbell (for female patients). The number of repetitions of dumbbell curls in 30 s was recorded. For the assessment of the sit to stand test, subjects were instructed to stand from a chair and then return to the sitting position five times. In addition, the physical performance of subjects was assessed by the Short Physical Performance Battery (SPPB), which included a balance test, gait speed test, and chair-stand test [29]. Sarcopenia and its diagnostic components were defined according to the Asian Working Group for Sarcopenia [30], including the value of CC < 34 cm for males or <33 cm for females defined as low CC, the value of ASMI < 7.0 kg/m^2^ for males or <5.4 kg/m^2^ for females defined as low ASMI, the handgrip strength <28 kg for males or <18 kg for females defined as low handgrip, five-time sit to stand test ≥12 s defined as poor sit to stand test, the gait speed <1.0 m/s defined as low gait speed, and the SPPB score ≤9 defined as low SPPB. Sarcopenic obesity was defined as the subject meets the diagnosis of sarcopenia and obesity (body fat percentage ≥25% for males or ≥30% for females).

### 2.6. Statistical Analyses

SigmaPlot software (version 12.0, SYSTAT, San Jose, CA, USA) was used to examine all statistical tests in the present study. The descriptive statistics are presented as the means  ±  standard deviation (medians) or numbers of participants (percentages). We used the Shapiro–Wilk test to examine the normality of the distribution of the data. The independent t-test or Mann–Whitney rank sum test was used to compare the difference in continuous variables between the mild dementia and moderate dementia groups. A chi-square test or Fisher’s exact test was used to examine the difference in categorical variables between the mild dementia and moderate dementia groups. The correlations between nutritional status, frailty, depression, quality of life, and muscle function were examined by Pearson correlation (a parametric test) or Spearman’s rank order correlation (a non-parametric test) coefficient based on the normal or non-normal distribution of the data. Multiple logistic regression was used to examine the associations between MNA scores and frailty, depression, quality of life, and sarcopenia after adjusting for age, gender, and exercise habit. The logistic regression model’s goodness of fit was evaluated by the value of Hosmer-Lemeshow test and calculate Nagelkerke R^2^ value, which was examined by using IBM SPSS Statistics for Windows (version 20.0, Armonk, New York, NY, USA). The statistical significance level was set at a *p* value <  0.05.

## 3. Results

### 3.1. Characteristics of Patients

The mean age of the participants was 76.5 ± 6.3 years, and the proportion of females was 72.5%. Table 1 shows the data on patient characteristics stratified by dementia severity. The patients with moderate dementia had significantly lower MMSE scores (*p* < 0.01); however, there was no significant difference in age, sex, blood pressure, biochemical data, education level, tobacco use, alcohol use, or exercise habits between patients with mild dementia and patients with moderate dementia. Although there was no significant difference in anthropometric data between the mild and moderate dementia groups, the values for waist circumferences in both genders and body fat percentage in females were higher than the reference values (waist, male < 90 cm, female < 80 cm; body fat, female < 30%). Additionally, there was a higher proportion of obesity in the moderate dementia group than in the mild dementia group (*p* = 0.09), which was slightly significant.

### 3.2. Nutritional Status, Frailty, Depression, Quality of Life, Muscle Mass, Strength and Endurance

Table 2 shows the scores for nutritional status, frailty, depression, and quality of life of patients with dementia. Patients with moderate dementia had a significantly lower MNA score (*p* < 0.01) and quality of life (*p* < 0.01) than those with mild dementia. In addition, the proportion of malnutrition (MNA < 24 points, *p* = 0.02) was higher in the patients with moderate dementia than in those with mild dementia. Regarding muscle mass, strength, and endurance, patients with moderate dementia had significantly lower values of CC (*p* < 0.01), whole body muscle mass (*p* = 0.01), ASMI (*p* = 0.01), handgrip strength (*p* < 0.01), dumbbell curls (*p* = 0.04), leg-back strength (*p* = 0.02), and SPPB (*p* = 0.02) than those with mild dementia.

### 3.3. The Sarcopenia Components of Patients

**Figure 1:**
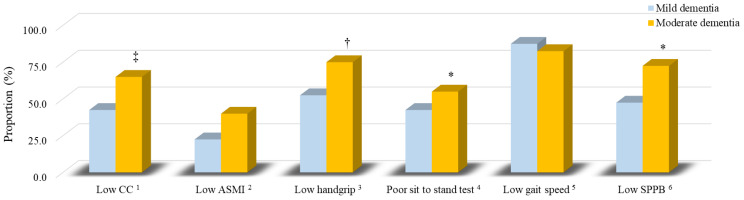
The proportion of sarcopenia components of patients with dementia. Blue is mild dementia; yellow is moderate dementia. ^1^ CC for male: <34 cm; female: <33 cm. ^2^ ASMI for male: <7.0 kg/m^2^; female: <5.4 kg/m^2^. ^3^ Handgrip strength for male: <28 kg; female: <18 kg. ^4^ Five-time sit to stand test ≥12 s. ^5^ The gait speed <1.0 m/s. ^6^ The SPPB score ≤9. *, *p* < 0.05; †, *p* = 0.06; ‡, *p* = 0.07. ASMI, appendicular skeletal muscle mass index; CC, calf circumference; SPPB, short physical performance battery.

Figure 1 shows the proportion of sarcopenia components in patients with dementia. Patients with moderate dementia had a significantly higher proportion of poor sit-to-stand test results (55.0% vs. 42.5%, *p* = 0.04) and low SPPB scores (72.5% vs. 47.5%, *p* = 0.04) than patients with mild dementia. The proportion of low handgrip strength (75.0% vs. 52.5%, *p* = 0.06) and low CC (65.0% vs. 42.5%, *p* = 0.07) was slightly higher in the patients with moderate dementia than in those with mild dementia.

### 3.4. Correlations between Nutrition Status and the Severity of Dementia, Frailty, Depression and Quality of Life

**Figure 2:**
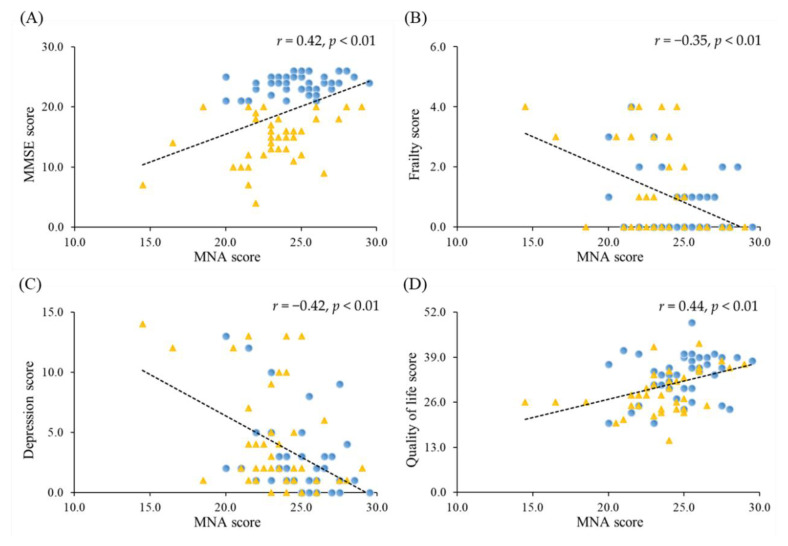
Correlations between nutritional status and the severity of dementia, frailty, depression, and quality of life. (**A**) Correlation between MNA and MMSE score (*r* = 0.42; *p* < 0.01). (**B**) Correlation between MNA and frailty score (*r* = −0.35; *p* < 0.01). (**C**) Correlation between MNA and depression score (*r* = −0.42; *p* < 0.01). (**D**) Correlation between MNA and quality of life score (*r* = 0.44; *p* < 0.01). Blue dot is mild dementia; yellow triangle is moderate dementia. MMSE, Mini Mental State Examination; MNA, mini nutritional assessment.

The correlations between MNA score and the severity of dementia, frailty, depression, and quality of life in patients with dementia are shown in Figure 2. The MNA score was significantly positively correlated with the MMSE (*r* = 0.42; *p* < 0.01) and quality of life (*r* = 0.44; *p* < 0.01) and significantly negatively correlated with frailty (*r* = −0.35; *p* < 0.01) and depression (*r* = −0.42; *p* < 0.01).

### 3.5. Correlations between Nutrition Status, Frailty, Depression, Quality of Life and Muscle Function

Table 3 shows the correlations between MNA score, frailty, depression, quality of life and muscle mass, strength, and endurance in patients with dementia. The MNA score was significantly positively correlated with muscle mass, muscle strength (handgrip strength), and endurance (dumbbell curls and gait speed) (*p* < 0.05). The frailty score was significantly correlated with muscle strength and endurance (*p* < 0.05). The depression and quality of life scores were significantly correlated with muscle endurance (*p* < 0.05).

### 3.6. Associations between Nutritional Status and Frailty, Depression, Quality of Life and Sarcopenia

Table 4 examines the associations between nutritional status and frailty, depression, quality of life, and sarcopenia. Patients with a lower MNA score were significantly associated with increased risk of frailty (odds ratio: 4.76, *p* < 0.01), depression (odds ratio: 3.17, *p* = 0.03), poor quality of life (odds ratio: 2.73, *p* < 0.05), low CC (odds ratio: 3.11, *p* = 0.02) and low handgrip (odds ratio: 2.52, *p* = 0.07). Additionally, after adjusting for potential confounders, a lower MNA score was significantly associated with an increased risk of low ASMI (odds ratio: 3.54, *p* = 0.04) and sarcopenia (odds ratio: 3.97, *p* = 0.03) in patients with dementia. In the adjusted regression models, the Hosmer and Lemeshow tests were all above 0.05, indicating that it was a goodness of *fit* test for logistic regression, and the values of Nagelkerke R^2^ for frailty, poor quality of life, low CC, low ASMI, and sarcopenia were 22.1%, 24.9%, 18.6%, 27.9%, and 29.8%, respectively. 

### 3.7. The Proportion of Sarcopenic Obesity in Patients with Dementia

**Figure 3:**
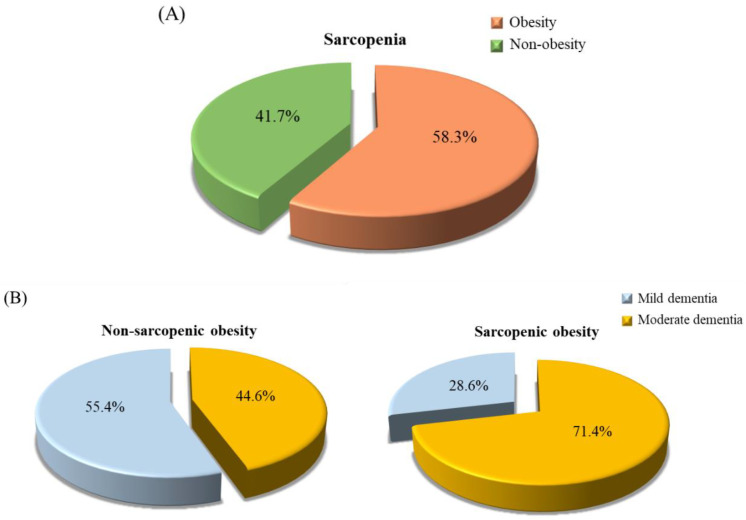
The proportion of sarcopenic obesity in patients with dementia. (**A**) The proportion of obesity in dementia patients with sarcopenia. Green is obesity dementia patients with sarcopenia; orange is non-obesity dementia patients with sarcopenia. (**B**) The proportion of dementia severity in patients with dementia stratified by sarcopenic obesity (*p* = 0.13). Blue is mild dementia; yellow is moderate dementia.

A total of twenty-four patients with sarcopenia in the present study. We further calculated the proportion of sarcopenic obesity in patients with dementia (Figure 3). Fifty-eight percent of dementia patients had sarcopenic obesity (Figure 3A). A total of 71.4% and 44.6% of patients had sarcopenic obesity in moderate and mild dementia, respectively (*p* = 0.13, Figure 3B).

## 4. Discussion

In the present study, we found that patients with dementia were suffering from malnutrition. Our study noted that patients with moderate dementia had a significantly lower MNA score (Table 2), 55% of patients with moderate dementia were at risk for malnutrition status (MNA < 24), and malnutrition was associated with the progression of the disease (Figure 2A). From the MNA questionnaire, we found that patients with dementia had a lower MNA score due to digestive problems, chewing or swallowing difficulties, weight loss during the last 3 months, neuropsychological problems, BMI < 19 kg/m^2^, and a lower intake of daily food such as dairy products, vegetables, and fruits (Supplementary Table S1). A previous study indicated that patients with dementia often face eating behavior disturbances, loss of independence, and depression, which may be predictors of malnutrition [31]. According to the report of the 2013–2016 National Nutrition Survey of Taiwan also noted that malnutrition is prevalent in the Taiwanese elderly [32]. Although the assessment of nutritional status that we used in the present study (MNA) is different from the National Nutrition Survey of Taiwan, the MNA nutritional assessment is widely used in long-term care and in clinical application. Likewise, patients with dementia who suffer from malnutrition may have faster cognitive decline and worse functional impairment in the progression of dementia [33]. Thus, we suggest that nutritional assessment should be a standard approach in patients with dementia (particular they are elderly) and that follow-up processes work to prevent malnutrition’s contribution to disease progression.

Patients with dementia face not only malnutrition but also the risk of sarcopenia. In our study, more than half of the patients with moderate dementia had low muscle mass, strength, and endurance (Figure 1). It is worth noting that the proportion of low gait speed in both mild and moderate dementia patients was very high (mild vs. moderate: 87.5% vs. 82.5%). In addition, the amount of muscle mass, strength and endurance were significantly lower with the progression of the disease (Table 2), which may be associated to nutritional status (Table 4). From the data of the nutritional status assessment questionnaire (MNA), we found that 76.3% of patients with dementia did not consume at least one serving of dairy products per day, 21.3% of patients did not consume meat, fish, or poultry every day, and 42.5% of patients did not consume two or more servings of fruit or vegetables per day (Supplementary Table S1), it reflects our patients may have insufficient intake of protein-rich food, especially from dairy. Recently, a systematic review and meta-analysis reported that total protein consumption was associated with body mass increase in a dose-response relationship [34]. As a result, we recommend that patients with dementia need to intake sufficient protein to sustain and improve their muscle mass. Additionally, it should be noted that up to 58.3% of dementia patients with sarcopenia were obese (Figure 3A), and 71.4% of patients with sarcopenic obesity had moderate dementia (Figure 3B). Based on the data (Figure 3), we theorize that the cause of malnutrition in patients with dementia is an unbalanced diet. In addition, as dementia is an aging-related disease, the basal metabolic rate and energy requirement may decrease in these elderly patients [35]. The volume of skeletal musculature decreases and the percentage of fat tissue increases with aging. In fact, we suspect that the risk of malnutrition from MNA in the present study was underestimated. The change in weight in the MNA questionnaire only assessed “weight loss” during the last three months. We suggest that the MNA include a scoring item for “weight gain”. Nutritional management should be integrated into dementia care [7]. A regular nutritional status assessment in patients with dementia may be useful in predicting the progression of the disease and may provide a target for clinical intervention.

Depression is a cause and consequence of dementia [36] and is associated with reduced quality of life [37] and increased risk of frailty [38]. In our study, we found that frailty, depression, and quality of life in patients with dementia were significantly related to nutritional status (Figure 2 and Table 4). Nutritional health is an essential component of quality of life in an aging population [39]. In our study, higher nutritional status was positively associated with muscle mass, strength, and endurance, and the muscle function was also related to frailty, depression, and quality of life in patients with dementia (Table 3). It is interesting to note that muscular endurance parameters, such as dumbbell curls, sit-stand tests, gait speed, and SPPB, were significantly associated with nutritional status, frailty, depression, and quality of life (Table 3). The muscle endurance may respond to the individual nutritional status and frail state, and further influence the quality of life, including mood state, particularly in older people [40,41]. A cross-sectional study that enrolled community-dwelling older adults in Taiwan also observed that physical fitness was related to quality of life [42]. In the elderly, declining muscle strength and endurance status have been found to further impact personal physical activity, negatively affecting depression and long-term quality of life [43,44]. Since dementia is an aging-related disease, assessing the muscle strength and endurance of patients with dementia should be included in dementia care in order to reduce the risk of frailty and depression and slow the decline in quality of life.

In the present study, a high prevalence of sarcopenia was found in dementia patients, with sarcopenia worsening as the disease progresses. We have demonstrated that nutritional status is related to the risk of sarcopenia, frailty, depression, and quality of life in patients with dementia. Most of the patients with dementia in this study suffered from sarcopenic obesity, which implies that dementia patients not only face skeletal musculature loss but also face accumulation of body fat. The limitations of this study include that the patients were from a single Taiwanese medical center, and a lack of a control group (without dementia) to compare, and we cannot clarify the causal relationship between nutritional status and dementia in this cross-sectional study. Second, the critical cause of dementia including Alzheimer’s disease or vascular dementia had not been confirmed. Although the small sample size was one of the limitations in the present study, we have tried to perform the post hoc tests to calculate the statistical power of the sample size and found the statistical power for correlations between MNA score and these outcomes such as muscle mass, frailty, depression, and quality of life have reached 0.89–0.99. Large-scale and case-control studies are needed to detect the differences in nutritional status between patients with dementia and non-dementia. Third, although we did not examine the reliability of the questionnaires (MNA, SOF, geriatric depression scale, and quality of life), these questionnaires were well developed and validated in clinical practice; besides, all questionnaires were performed by the research assistant from asked the subjects or their caregiver carefully to reduce the reported error. Regarding the reliability of the questionnaires, these questionnaires detected similar domains, such as body weight changes, activity, and mood states. We attempted to examine the consistency of MNA, SOF, GDS, and, QOL on similar questions and we found the consistency for the response status of the cases were high (>90%). So, the response status of our cases in the present was consistent. 

## 5. Conclusions

In patients with dementia, the risk of malnutrition was significantly correlated with muscle function, as well as the risk of sarcopenia, frailty, depression, and decreased quality of life, all of which worsen with the severity of the disease. Thus, we suggest that in patients with dementia, nutritional status should be monitored regularly to prevent the risk of sarcopenia and improve quality of life along with slowing the progression of dementia. Lifestyle interventions, including caloric restriction, protein supplementation, and physical activity consisting of aerobic and resistance exercise, have been considered the cornerstone of the treatment of sarcopenic obesity [19]. Thus, future studies should clarify the effect of nutritional intervention on the risk of sarcopenic obesity in patients with dementia.

## Figures and Tables

**Table 1:** Characteristics of patients with dementia.

	Mild Dementia (N = 40)	Moderate Dementia (N = 40)	*p* Value
MMSE score	23.9 ± 1.6 (24.0)	14.6 ± 4.1 (15.0)	<0.01
Age (years)	76.0 ± 6.4 (76.0)	76.9 ± 6.2 (78.0)	0.54
Female (n, %)	26 (65.0%)	32 (80.0%)	0.21
BMI (kg/m^2^)	24.6 ± 3.8 (24.3)	23.8 ± 3.9 (23.5)	0.32
Waist (cm)	91.9 ± 9.5 (92.3)	89.5 ± 10.4 (89.0)	0.29
Male	91.9 ± 8.3 (92.8)	90.4 ± 4.9 (90.0)	0.66
Female	91.9 ± 10.3 (92.0)	89.3 ± 11.4 (86.8)	0.37
Body fat (%)	28.4 ± 7.5 (29.1)	30.5 ± 5.4 (30.9)	0.17
Male	22.3 ± 6.5 (23.0)	24.0 ± 3.2 (24.4)	0.48
Female	31.8 ± 5.7 (31.8)	32.1 ± 4.5 (32.2)	0.80
Obesity (n, %) ^1^	18 (45.0%)	26 (65.0%)	0.09
Male	3 (21.4%)	4 (50.0%)	0.34
Female	15 (57.7%)	22 (68.8%)	0.44
SBP (mmHg)	130.8 ± 17.2 (131.0)	128.4 ± 17.9 (129.5)	0.53
DBP (mmHg)	74.1 ± 10.0 (72.5)	71.9 ± 10.7 (72.0)	0.34
Albumin (g/L)	45.0 ± 2.0 (45.0)	44.0 ± 3.0 (44.0)	0.16
Fasting glucose (mmol/L)	6.4 ± 1.4 (6.1)	6.7 ± 1.8 (6.1)	0.84
Total cholesterol (mmol/L)	4.9 ± 0.9 (4.7)	4.8 ± 1.0 (4.5)	0.45
GPT (U/L)	20.3 ± 13.1 (16.5)	19.1 ± 7.9 (18.5)	0.71
Creatinine (µmol/L)	75.1 ± 15.9 (73.4)	79.6 ± 23.9 (71.6)	0.64
Education			0.16
None	4 (10.0%)	11 (27.5%)	
Elementary school	18 (45.0%)	15 (37.5%)	
Junior high school	8 (20.0%)	7 (17.5%)	
Senior high school	7 (17.5%)	7 (17.5%)	
University or above	3 (7.5%)	0 (0.0%)	
Tobacco use	4 (10.0%)	6 (15.0%)	0.74
Alcohol use	3 (7.5%)	5 (12.5%)	0.71
Exercise habit ^2^	26 (65.0%)	21 (52.5%)	0.36
Family history of dementia (n, %)	9 (22.5%)	5 (12.5%)	0.57

Data was presented as means ± SD (medians). ^1^ A body fat percentage ≥25% in males and ≥30% in females. ^2^ Exercise habit was defined as patients with regular exercise at least 3 times a week. BMI, body mass index; DBP, diastolic blood pressure; GPT, glutamic pyruvic transaminase; SBP, systolic blood pressure.

**Table 2:** Nutritional status, frailty, depression, quality of life, and muscle mass, strength, and endurance of patients with dementia.

	Mild Dementia (N = 40)	Moderate Dementia (N = 40)	*p* Value
MNA score (points)	24.9 ± 2.2 (25.0)	23.3 ± 2.8 (23.5)	<0.01
MNA < 24 (n, %)	11 (27.5%)	22 (55.0%)	0.02
Frailty score (points)	0.75 ± 1.08 (0.00)	1.30 ± 1.52 (1.00)	0.15
Frailty ≥ 2 (n, %)	8 (20.0%)	14 (35.0%)	0.21
Depression score (points)	2.8 ± 3.3 (2.0)	4.3 ± 4.5 (2.0)	0.11
Depression ≥ 5 (n, %)	8 (20.0%)	13 (32.5%)	0.31
Quality of life score (points)	33.6 ± 6.5 (35.0)	28.8 ± 5.9 (28.0)	<0.01
Quality of life ≤ 26 (n, %)	8 (20.0%)	16 (40.0%)	0.09
Muscle mass			
CC (cm)			
Overall	33.9 ± 2.8 (33.8)	32.0 ± 2.7 (32.0)	<0.01
Male	34.4 ± 2.5 (34.3)	32.5 ± 2.5 (32.3)	0.10
Female	33.6 ± 3.0 (33.6)	31.9 ± 2.8 (31.8)	0.03
Whole body muscle mass (kg)			
Overall	40.1 ± 6.9 (39.9)	36.3 ± 6.4 (34.7)	0.01
Male	46.4 ± 4.3 (47.0)	45.0 ± 3.5 (45.1)	0.42
Female	36.7 ± 5.4 (36.9)	34.1 ± 4.9 (33.9)	0.06
ASMI (kg/m^2^)			
Overall	6.7 ± 0.9 (6.8)	6.1 ± 1.1 (6.1)	0.01
Male	7.2 ± 0.6 (7.3)	6.8 ± 0.6 (6.8)	0.16
Female	6.5 ± 0.9 (6.5)	6.0 ± 1.1 (5.8)	0.08
Muscle strength			
Handgrip strength (kg)			
Overall	20.6 ± 7.6 (20.5)	16.1 ± 6.9 (16.8)	<0.01
Male	28.6 ± 4.1 (27.8)	22.0 ± 9.2 (25.3)	0.03
Female	16.2 ± 5.0 (17.3)	14.6 ± 5.4 (15.6)	0.24
Leg-back strength (kg)			
Overall	45.3 ± 22.8 (43.0)	34.9 ± 18.6 (28.5)	0.02
Male	67.8 ± 20.4 (65.3)	53.3 ± 28.9 (51.0)	0.18
Female	33.2 ± 12.5 (27.5)	30.1 ± 11.1 (26.0)	0.29
Muscle endurance			
Dumbbells curls (reps)			
Overall	13.8 ± 6.1 (14.0)	11.0 ± 7.7 (10.0)	0.04
Male	16.4 ± 7.2 (18.0)	13.8 ± 12.7 (15.0)	0.53
Female	12.4 ± 5.0 (12.0)	10.3 ± 5.9 (10.0)	0.15
Sit to stand test (s)			
Overall	13.6 ± 5.6 (11.0)	18.2 ± 10.8 (16.0)	0.09
Male	12.7 ± 5.1 (10.5)	12.7 ± 8.9 (10.5)	0.59
Female	14.1 ± 6.0 (11.5)	19.7 ± 11.0 (17.0)	0.06
Gait speed (m/s)			
Overall	0.72 ± 0.24 (0.71)	0.66 ± 0.22 (0.67)	0.26
Male	0.77 ± 0.28 (0.72)	0.83 ± 0.24 (0.80)	0.65
Female	0.69 ± 0.22 (0.71)	0.61 ± 0.19 (0.67)	0.19
SPPB (scores)			
Overall	9.3 ± 2.7 (10.0)	7.2 ± 3.9 (8.5)	0.02
Male	10.1 ± 2.1 (10.5)	8.4 ± 4.5 (10.0)	0.55
Female	8.8 ± 2.9 (9.0)	6.9 ± 3.7 (8.0)	0.08

Data was presented as means ± SD (medians). ASMI, appendicular skeletal muscle mass index; CC, calf circumference; MMSE, Mini Mental State Examination; MNA, mini nutritional assessment; SPPB, short physical performance battery.

**Table 3:** Correlations between nutritional status, frailty, depression, quality of life and muscle function in patients with dementia.

	MNA Score	Frailty Score	Depression Score	Quality of Life Score
	*R*^1^ (*p* Value)			
Muscle mass				
CC (cm)	0.45 (<0.01)	−0.07 (0.56)	−0.21 (0.06)	0.17 (0.14)
Whole body muscle mass (kg)	0.35 (<0.01)	0.10 (0.36)	−0.14 (0.23)	−0.10 (0.40)
ASMI (kg/m^2^)	0.36 (<0.01)	0.06 (0.60)	−0.11 (0.32)	0.00 (1.00)
Muscle strength				
Handgrip strength (kg)	0.26 (0.02)	−0.27 (0.02)	−0.19 (0.10)	0.04 (0.75)
Leg-back strength (kg)	0.10 (0.40)	−0.35 (<0.01)	−0.18 (0.11)	0.06 (0.61)
Muscle endurance				
Dumbbells curls (reps)	0.25 (0.03)	−0.62 (<0.01)	−0.44 (<0.01)	0.35 (<0.01)
Sit to stand test (s)	−0.17 (0.17)	0.48 (<0.01)	0.43 (<0.01)	−0.31 (0.01)
Gait speed (m/s)	0.24 (0.04)	−0.43 (<0.01)	−0.28 (0.01)	0.17 (0.15)
SPPB (scores)	0.16 (0.15)	−0.62 (<0.01)	−0.34 (<0.01)	0.32 (<0.01)

^1^ Correlation coefficient. ASMI, appendicular skeletal muscle mass index; CC, calf circumference; MNA, mini nutritional assessment; SPPB, short physical performance battery.

**Table 4:** Associations between nutritional status and frailty, depression, quality of life, and sarcopenia.

	Poor MNA ^1^				
	Unadjusted		Adjusted ^12^		
	Odds Ratios (95% CI)	*p* Value	Odds Ratios (95% CI)	*p* Value	Hosmer-Lemeshow Test Pr > Chi-Square
Normal MNA ^1^	1.00	-	1.00	-	
Frailty ^2^	4.76 (1.66–13.69)	<0.01	4.35 (1.41–13.39)	0.01	0.75
Depression ^3^	3.17 (1.13–8.90)	0.03	2.62 (0.86–8.00)	0.09	0.75
Poor quality of life ^4^	2.73 (1.02–7.28)	<0.05	3.27 (1.04–10.33)	0.04	0.37
Sarcopenia components					
Low CC ^5^	3.11 (1.21–7.96)	0.02	4.00 (1.39–11.45)	0.01	0.92
Low ASMI ^6^	2.00 (0.76–5.23)	0.16	3.54 (1.09–11.53)	0.04	0.18
Low handgrip ^7^	2.52 (0.95–6.74)	0.07	2.42 (0.82–7.18)	0.11	0.79
Poor sit to stand test ^8^	1.74 (0.61–4.95)	0.30	1.49 (0.50–4.43)	0.47	0.97
Low gait speed ^9^	1.32 (0.23–7.70)	0.76	0.30 (0.02–3.88)	0.36	0.96
Low SPPB ^10^	2.02 (0.79–5.17)	0.14	1.61 (0.57–4.58)	0.37	0.56
Sarcopenia ^11^	2.24 (0.84–5.95)	0.11	3.97 (1.19–13.27)	0.03	0.63

^1^ Poor MNA: MNA score <24; Normal MNA: MNA score ≥24. ^2^ Frailty: frailty score ≥2. ^3^ Depression: depression score ≥5. ^4^ Poor quality of life, quality of life score ≤26. ^5^ CC for male: <34 cm; female: <33 cm. ^6^ ASMI for male: <7.0 kg/m^2^; female: <5.4 kg/m^2^. ^7^ Handgrip strength for male: <28 kg; female: <18 kg. ^8^ Five-time sit to stand test ≥12 s. ^9^ The gait speed <1.0 m/s. ^10^ The SPPB score ≤9. ^11^ Low ASMI and low muscle strength and endurance according to the Asian Working Group for Sarcopenia. ^12^ Adjusted for age, gender, and exercise habit. ASMI, appendicular skeletal muscle mass index; CC, calf circumference; CI, confidence interval; MNA, mini nutritional assessment; SPPB, short physical performance battery.

## Data Availability

The datasets generated and/or analyzed during the current study are available from the corresponding author on reasonable request.

## Supplementary Materials

The following are available online at https://www.mdpi.com/article/10.3390/ijerph19052492/s1. Table S1: The response of Mini Nutritional Assessment (MNA) questionnaire in patients with dementia.

## References

[1] World Health Organization The Top 10 Causes of Death. https://www.who.int/news-room/fact-sheets/detail/the-top-10-causes-of-death.

[2] Baumgart M., Snyder H.M., Carrillo M.C., Fazio S., Kim H., Johns H. (2015). Summary of the evidence on modifiable risk factors for cognitive decline and dementia: A population-based perspective. Alzheimers Dement..

[3] Lin Y.Y., Huang C.S. (2016). Aging in Taiwan: Building a Society for Active Aging and Aging in Place. Gerontologist.

[4] Taiwan Alzheimer Disease Association People with Dementia in Taiwan. (April 2021). http://www.tada2002.org.tw/About/IsntDementia#bn1.

[5] Scott K.R., Barrett A.M. (2007). Dementia syndromes: Evaluation and treatment. Expert Rev. Neurother..

[6] Cortes F., Nourhashémi F., Guérin O., Cantet C., Gillette-Guyonnet S., Andrieu S., Ousset P.J., Vellas B., REAL-FR Group (2008). Prognosis of Alzheimer’s disease today: A two-year prospective study in 686 patients from the REAL-FR Study. Alzheimers Dement..

[7] Volkert D., Chourdakis M., Faxen-Irving G., Frühwald T., Landi F., Suominen M.H., Vandewoude M., Wirth R., Schneider S.M. (2015). ESPEN guidelines on nutrition in dementia. Clin. Nutr..

[8] Larsson L., Degens H., Li M., Salviati L., Lee Y.I., Thompson W., Kirkland J.L., Sandri M. (2019). Sarcopenia: Aging-Related Loss of Muscle Mass and Function. Physiol. Rev..

[9] Nishimoto Y., Arai Y. (2018). Aging-related frailty and sarcopenia. Frailty and sarcopenia in the centenarians. Clin. Calcium.

[10] Matsumoto H., Tanimura C., Tanishima S., Osaki M., Noma H., Hagino H. (2017). Sarcopenia is a risk factor for falling in independently living Japanese older adults: A 2-year prospective cohort study of the GAINA study. Geriatr. Gerontol. Int..

[11] Yeung S.S.Y., Reijnierse E.M., Pham V.K., Trappenburg M.C., Lim W.K., Meskers C.G.M., Maier A.B. (2019). Sarcopenia and its association with falls and fractures in older adults: A systematic review and meta-analysis. J. Cachexia Sarcopenia Muscle.

[12] Kitamura A., Seino S., Abe T., Nofuji Y., Yokoyama Y., Amano H., Nishi M., Taniguchi Y., Narita M., Fujiwara Y. (2021). Sarcopenia: Prevalence; associated factors; and the risk of mortality and disability in Japanese older adults. J. Cachexia Sarcopenia Muscle.

[13] Crocker T.F., Brown L., Clegg A., Farley K., Franklin M., Simpkins S., Young J. (2019). Quality of life is substantially worse for community-dwelling older people living with frailty: Systematic review and meta-analysis. Qual. Life Res..

[14] Gobbens R.J.J., van Assen M.A.L.M. (2017). Associations between multidimensional frailty and quality of life among Dutch older people. Arch. Gerontol. Geriatr..

[15] Cabett Cipolli G., Sanches Yassuda M., Aprahamian I. (2019). Sarcopenia Is Associated with Cognitive Impairment in Older Adults: A Systematic Review and Meta-Analysis. J. Nutr. Health Aging.

[16] Chang K.V., Hsu T.H., Wu W.T., Huang K.C., Han D.S. (2016). Association Between Sarcopenia and Cognitive Impairment: A Systematic Review and Meta-Analysis. J. Am. Med. Dir. Assoc..

[17] Peng T.C., Chen W.L., Wu L.W., Chang Y.W., Kao T.W. (2020). Sarcopenia and cognitive impairment: A systematic review and meta-analysis. Clin. Nutr..

[18] Choi K.M. (2016). Sarcopenia and sarcopenic obesity. Korean J. Intern. Med..

[19] Batsis J.A., Villareal D.T. (2018). Sarcopenic obesity in older adults: Aetiology, epidemiology and treatment strategies. Nat. Rev. Endocrinol..

[20] Ministry of Health and Welfare Dementia Diagnostic Manual. (February 2017). https://www.mohw.gov.tw/dl-27189-8993c3ad-0f47-45e0-a602-6a4362faae9a.html.

[21] Folstein M.F., Folstein S.E., McHugh P.R. (1975). “Mini-mental state”. A practical method for grading the cognitive state of patients for the clinician. J. Psychiatr. Res..

[22] Nolan J.M., Mulcahy R., Power R., Moran R., Howard A.N. (2018). Nutritional Intervention to Prevent Alzheimer’s Disease: Potential Benefits of Xanthophyll Carotenoids and Omega-3 Fatty Acids Combined. J. Alzheimers Dis..

[23] Okorodudu D.O., Jumean M.F., Montori V.M., Romero-Corral A., Somers V.K., Erwin P.J., Lopez-Jimenez F. (2010). Diagnostic performance of body mass index to identify obesity as defined by body adiposity: A systematic review and meta-analysis. Int. J. Obes..

[24] Vellas B., Villars H., Abellan G., Soto M.E., Rolland Y., Guigoz Y., Morley J.E., Chumlea W., Salva A., Rubenstein L.Z. (2006). Overview of the MNA—Its history and challenges. J. Nutr. Health Aging.

[25] Ensrud K.E., Ewing S.K., Taylor B.C., Fink H.A., Cawthon P.M., Stone K.L., Hillier T.A., Cauley J.A., Hochberg M.C., Rodondi N. (2008). Comparison of 2 frailty indexes for prediction of falls; disability; fractures; and death in older women. Arch. Intern. Med..

[26] Yesavage J.A., Sheikh J.I. (1986). Geriatric Depression Scale (GDS): Recent evidence and development of a shorter version. Clin. Gerontol. J. Aging Ment. Health.

[27] Logsdon R.G., Gibbons L.E., McCurry S.M., Teri L. (1999). Quality of life in Alzheimer’s disease: Patient and caregiver reports. J. Ment. Health Aging.

[28] Rikli R.E., Jones C.J. (2013). Senior Fitness Test Manual. Champaign IL Hum. Kinet..

[29] Guralnik J.M., Simonsick E.M., Ferrucci L., Glynn R.J., Berkman L.F., Blazer D.G., Scherr P.A., Wallace R.B. (1994). A short physical performance battery assessing lower extremity function: Association with self-reported disability and prediction of mortality and nursing home admission. J. Gerontol..

[30] Chen L.K., Woo J., Assantachai P., Auyeung T.W., Chou M.Y., Iijima K., Jang H.C., Kang L., Kim M., Kim S. (2020). Asian Working Group for Sarcopenia: 2019 Consensus Update on Sarcopenia Diagnosis and Treatment. J. Am. Med. Dir. Assoc..

[31] Roqué M., Salvà A., Vellas B. (2013). Malnutrition in community-dwelling adults with dementia (NutriAlz Trial). J. Nutr. Health Aging.

[32] Ministry of Health and Welfare 2013–2016 National Nutrition Survey in Taiwan. (12 July 2019). https://www.hpa.gov.tw/Pages/Detail.aspx?nodeid=3999&pid=11145.

[33] Sanders C., Behrens S., Schwartz S., Wengreen H., Corcoran C.D., Lyketsos C.G., Tschanz J.T. (2016). Nutritional Status is Associated with Faster Cognitive Decline and Worse Functional Impairment in the Progression of Dementia: The Cache County Dementia Progression Study1. J. Alzheimers Dis..

[34] Tagawa R., Watanabe D., Ito K., Ueda K., Nakayama K., Sanbongi C., Miyachi M. (2020). Dose-response relationship between protein intake and muscle mass increase: A systematic review and meta-analysis of randomized controlled trials. Nutr. Rev..

[35] Shimokata H., Kuzuya F. (1993). Aging; basal metabolic rate; and nutrition. Nihon Ronen Igakkai Zasshi.

[36] Bennett S., Thomas A.J. (2014). Depression and dementia: Cause; consequence or coincidence?. Maturitas.

[37] Aziz R., Steffens D.C. (2013). What are the causes of late-life depression?. Psychiatr. Clin. N. Am..

[38] Soysal P., Veronese N., Thompson T., Kahl K.G., Fernandes B.S., Prina A.M., Solmi M., Schofield P., Koyanagi A., Tseng P.T. (2017). Relationship between depression and frailty in older adults: A systematic review and meta-analysis. Ageing Res. Rev..

[39] Bailly N., Maître I., Van Wymelbeke V. (2015). Relationships between nutritional status; depression and pleasure of eating in aging men and women. Arch. Gerontol. Geriatr..

[40] Engelheart S., Andrén D., Repsilber D., Bertéus F.H., Brummer R.J. (2021). Nutritional status in older people—An explorative analysis. Clin. Nutr. ESPEN.

[41] Tay L.B., Chua M.P., Tay E.L., Chan H.N., Mah S.M., Latib A., Wong C.Q., Ng Y.S. (2019). Multidomain Geriatric Screen and Physical Fitness Assessment Identify Prefrailty/Frailty and Potentially Modifiable Risk Factors in Community-Dwelling Older Adults. Ann. Acad. Med. Singap..

[42] Li P.S., Hsieh C.J., Miao N.F. (2020). A Study of Physical Activity; Frailty; and Health-Related Quality of Life Among Community-Dwelling Older Adults in Taiwan. J. Nurs. Res..

[43] Groessl E.J., Kaplan R.M., Rejeski W.J., Katula J.A., Glynn N.W., King A.C., Anton S.D., Walkup M., Lu C.J., Reid K. (2019). Physical Activity and Performance Impact Long-term Quality of Life in Older Adults at Risk for Major Mobility Disability. Am. J. Prev. Med..

[44] Sjöberg L., Karlsson B., Atti A.R., Skoog I., Fratiglioni L., Wang H.X. (2017). Prevalence of depression: Comparisons of different depression definitions in population-based samples of older adults. J. Affect. Disord..

