# Supplementary material for: Sarcopenic Obesity Tendency and Nutritional Status Is Related to the Risk of Sarcopenia, Frailty, Depression and Quality of Life in Patients with Dementia
Source: Int J Environ Res Public Health. 2022 Feb 22;19(5):2492. doi: 10.3390/ijerph19052492 (PMC8908993; doi:10.3390/ijerph19052492)
Supplement: Supplementary file 1 [file ijerph-19-02492-s001.zip › ijerph-1573832-supplementary.pdf]

International Journal of
Environmental Research
and Public Health

MDPI

Table S1. The response of Mini Nutritional Assessment (MNA) questionnaire in patients with dementia.

	Dementia (n = 80)
A1. Has moderate or severe food intake declined over the past 3 months due to loss of appetite, digestive problems, chewing or swallowing difficulties.	6 (7.5%)
A2. Weight loss during the last 3 months.	6 (7.5%)
A3. Mobility for bed or chair bound, or able to get out of bed / chair but does not go out.	2 (2.5%)
A4. Has suffered psychological stress or acute disease in the past 3 months.	0 (0.0%)
A5. Has suffered Neuropsychological problems.	70 (87.5%)
A6. Body mass index less than 19.	3 (3.8%)
A7. Lives dependently.	2 (2.5%)
A8. Takes more than 3 prescription drugs per day.	65 (81.3%)
A9. Has suffered pressure sores or skin ulcers.	2 (2.5%)
A10. Lower than 3 full meals for the patient eat daily.	10 (12.5%)
A11.1. No selected consumption markers for protein intake: at least one serving of dairy products, such as milk, cheese, yoghurt per day.	61 (76.3%)
A11.2. No selected consumption two or more servings of legumes or eggs per week.	11 (13.8%)
A11.3. No selected consumption meat, fish, or poultry every day.	17 (21.3%)
A12. No selected consumes two or more servings of fruit or vegetables per day.	34 (42.5%)
A13. Lower than 5 cups fluid (water, juice, coffee, tea, milk...) is consumed per day.	55 (68.8%)
A14. Unable to eat without assistance or self-fed with some difficulty.	2 (2.5%)
A15. Views self as being malnourished.	5 (6.3%)
A16. Not feel good for health status in comparison with other people of the same age.	11 (13.8%)
A17. Mid-arm circumference less than 22 cm.	1 (1.3%)
A18. Calf circumference less than 31 cm.	16 (20.0%)

	MNA ≥ 24 (n = 47)	MNA < 24 (n = 33)	*p* values
A1. Has moderate or severe food intake declined over the past 3 months due to loss of appetite, digestive problems, chewing or swallowing difficulties.	1 (2.1%)	5 (15.2%)	0.08
A2. Weight loss during the last 3 months.	3 (6.4%)	3 (9.1%)	< 0.05
A3. Mobility for bed or chair bound, or able to get out of bed / chair but does not go out.	0 (0.0%)	2 (6.1%)	0.17
A4. Has suffered psychological stress or acute disease in the past 3 months.	0 (0.0%)	0 (0.0%)	-
A5. Has suffered Neuropsychological problems.	37 (78.7%)	33 (100.0%)	< 0.01
A6. Body mass index less than 19.	0 (0.0%)	3 (9.1%)	0.07
A7. Lives dependently.	0 (0.0%)	2 (6.1%)	0.17
A8. Takes more than 3 prescription drugs per day.	40 (85.1%)	25 (75.8%)	0.45
A9. Has suffered pressure sores or skin ulcers.	2 (4.3%)	0 (0.0%)	0.51
A10. Lower than 3 full meals for the patient eat daily.	4 (8.5%)	6 (18.2%)	0.30
A11.1. No selected consumption markers for protein intake: at least one serving of dairy products, such as milk, cheese, yoghurt per day.	34 (72.3%)	27 (81.8%)	0.48
A11.2. No selected consumption two or more servings of legumes or eggs per week.	4 (8.5%)	7 (21.2%)	0.19
A11.3. No selected consumption meat, fish, or poultry every day.	7 (14.9%)	10 (30.3%)	0.17
A12. No selected consumes two or more servings of fruit or vegetables per day.	8 (17.0%)	26 (78.8%)	< 0.01
A13. Lower than 5 cups fluid (water, juice, coffee, tea, milk...) is consumed per day.	26 (55.3%)	29 (87.9%)	< 0.01
A14. Unable to eat without assistance or self-fed with some difficulty.	2 (4.3%)	0 (0.0%)	0.51
A15. Views self as being malnourished.	0 (0.0%)	5 (15.2%)	< 0.01
A16. Not feel good for health status in comparison with other people of the same age.	4 (8.5%)	7 (21.2%)	< 0.01
A17. Mid-arm circumference less than 22 cm.	0 (0.0%)	1 (3.0%)	0.41
A18. Calf circumference less than 31 cm.	4 (8.5%)	12 (36.4%)	< 0.01
